# Pathological Relations of the Mouth

**Published:** 1849-10

**Authors:** W. R. Handy


THE AMERICAN

### Journal of Dental Science.

Vol. X.] OCTOBER, 1849. [No. 1.

### ARTICLE I.

*Pathological Relations of the Mouth.* By W. R. Handy, M. D.

In a previous number of the Journal we attempted to give the *anatomical and physiological relations of the mouth.* It was then stated, that the body consisted of a community of organs, each having a special and definite function, distinct and necessary for the common good of the whole, and that the presence and healthful action of the whole was as equally necessary for the proper integrity of each. In other words, the different parts or organs comprising the body, though varying ever so much in their forms, size, situation, structure, and functions, were, nevertheless, dependent the one upon the other—each upon the whole, and the whole upon each, for the common good of all, as well as the individual health of every separate part; thus forming a bond of union and reciprocal action, constituting the *normal* or *healthy relations* of the body—and that as the mouth constituted a part and belonged to this great and extensive family of organs, its relationship was consequently of so close and intimate a character, as to make it necessarily take part in, and to influence, and be influenced, by every other part, and in the precise proportion of the degree of its relation with these several parts, and thus sharing in every thing, involving the common health of the whole.

On the present occasion we propose examining, as briefly as possible, the *pathological relations* of the *mouth*, or the mouth in the state of disease; and inquire whether its relations in this condition are not equally as extensive and important as those it has in the state of health?

It is admitted by all, and conceded to follow as an irresistible consequence, that if all the organs and all their functions have an inseparable connection and dependency each with the other, and the whole with each, in the state of health, and that if the preservation of this health depends upon the continuance of this reciprocal relation between the several organs separately, as well as the whole collectively, we say, in this state of things, the proposition is at once conceded, that when this healthy relation, among the several organs, is disturbed or broken by disease in any one, that all the rest must, necessarily, suffer, in a greater or less degree, if the crippled condition of the organ be not corrected—and that the amount of this suffering, it will also be admitted, must also bear a like proportion to the relative amount of influence, which the diseased organ has with every other, in its physiological state, in preserving the common health of all.

Though this admission be made, yet we respectfully ask if it be not an admission more of *abstraction*, than one of *practical* reality? Is it carried out in the every day business of those professions whose glory consists in being guided by *principles* and not by *empiricism*?

To answer these questions, with a special application to the dental profession, will comprise most of the remarks we shall have to offer.

The *pathological relations* of the mouth comprehends two divisions, viz.

1st—General.

2nd—Special.

The first division comprises all those general relations of the body, connecting each and every organ into one common whole, and especially engaging the attention of the *physician* and *general surgeon.* While the second division is made to embrace all those diseased actions which belong to the several parts constituting the mouth, forming its special relations and consigned by common consent to the particular charge of the surgeon dentist.

Hence we see two very large and influential professions, viz. that of the physician and the dentist—deriving their origin and distinctiveness, simply by a separation of these general and special relations of the body—viewing them, for all practical purposes, as separate and independent, the one of the other, and hence making a divorce between the two, nowhere authorised by nature; but, on the contrary, are positive violations of her laws, which indissolubly unite these two classes of relations, as indispensably necessary to the health of the whole and every part of the body, and must, of consequence, be as indissolubly cemented together, and as necessarily taken into the account in every case of their practical restoration, whether attempted by physician or dentist, should either desire science, skill, or success to attend his efforts.

It is an unfortunate but nevertheless notorious fact, how widely separated these two kinds of relations have become, and what an independent and unnatural attitude they have been made to assume towards each other, in their practical application—by both of the two classes of professions just mentioned. In consequence of which, we think it may be safely asserted, that an immense amount of accumulated suffering has occurred, which otherwise might have been avoided: the true character and seat of many diseases have remained undetected, and their successful treatment, as a necessary consequence, an utter failure. So true is this assertion, that it is only necessary to state here, in general terms, as we intend in another place to present some cases in proof, that it is known to every well informed physician and dentist, that cases have occurred and are recorded, where patients have, year after year, taken medicine of every kind, and from the hands of the greatest variety of physicians, and all without avail—that such class of patients have, further, by the advice of their medical attendants, gone in pursuit of health and for the removal of their diseases, pronounced to be beyond the reach of medical and surgical skill—we say have gone, by the best medical advice, for the cure of their several cases, to distant regions, crossing seas, scaling mountains, visiting springs, taking every variety of exercise, and every variety of diet, and yet all of no avail—as they have severally returned home with little or no improvement in their disease, which still exhibits the same character and unmanagebleness as when they started on their tour. By some lucky or accidental suggestion, the *teeth* are suspected as the probable cause of the whole mischief, and with some degree of plausibility, as every thing else had failed to give any relief.

The suggestion thus, is acted upon—skillful dentists are employed, and by correcting the diseased condition of the teeth and mouth, the before incurable diseases of each become readily curable—the general health is speedily restored, and the whole of this wonderful change brought about simply by the application of a little dental skill.

In other words, disease had commenced in the mouth, and extended to other parts of the system—the general relations of the body became disturbed, and the physician, by attending solely to these latter, whose distubance was only secondary, and overlooking the special relations of the mouth, which was the source and cause of all the mischief.—We say by thus disregarding this law of connection established between these two classes of relations, and not giving the proper estimate to the reciprocal amount of influence which each exerts upon the other, when diseased, is the only explanation, we think, which can be fairly given for the failure in the diseases under notice—for when the cause was discovered, and the laws of pathological relationship obeyed, they were found to be perfectly manageable.

And here the question obtrudes itself, and most especially upon the general and unprofessional reader, viz. how is it possible that the great mass of physicians, and among those many of our best educated and most scientific men, could be guilty of such an oversight, in not at all, or but very slightly, looking to the mouth, as they do with all the rest of the body, in their usual most searching examinations for the seat and nature of every disease? The question is asked, how is this accounted for, and why is it so? The only satisfactory explanation, it seems to us, is found in the fact, that very few medical colleges spend much if any time in investigating the diseases of the mouth and the dental apparatus proper; so that the student graduates and enters the profession, not only with scarcely any knowledge of even the existence and character of dental diseases, but further, is strongly impressed with the dangerous idea, that a knowledge of such diseases must be of little value, and their influence in the causation of disease in every other part of the system, must be of the most trifling importance, since they scarcely ever heard any of the professors even mention such diseases in any of their lectures. It does not, therefore, appear so strange, that physicians, entering practice with such impressions and opinions, should fail to discover the cause of disease, when seated in the mouth; since their attention has never been directed to such a source, as being capable, by its pathological relations with the rest of the body, to justify even the suspicion, much less to have their attention seriously directed to such a quarter, as the origin of disease elsewhere located.

The authority of Dr. Rush on this point, though he invites attention to the teeth and mouth, as the occasional cause of disease elsewhere, is, nevertheless, conclusive as to the small amount of consideration which is in general bestowed upon this subject by physicians, in comparison with all others. His language is, "When we consider how often the teeth, when decayed, are exposed to irritation from hot and cold drinks, and aliments, and from pressure by mortification, and from cold air, and how intricate the connection of the mouth is with the whole system, I am disposed to believe they are often unsuspected causes of general, and particularly of nervous diseases. When we add to the list of these diseases, the morbid effects of the acrid and putrid matters which are sometimes discharged from carious teeth, or from ulcers in the gums, created by them, also the influence which both have in preventing perfect mastication, and the connection of that animal function with good health, I cannot help thinking that our success in the treatment of all chronic diseases would be very much promoted by directing our inquiries into the state of the teeth in sick people, and by advising their extraction in every case in which they are decayed."

If we now turn to the practical dentist, we will find that the general and special relations of the mouth are still more widely separated than is done by the physician—for, with the exception of many honorable and scientific dentists, it is a notorious fact that the great mass of this profession can lay no claims whatever to any scientific knowledge or skill in their calling—that they are most profoundly ignorant of even the simplest rudiments of anatomy and physiology—have about the same idea of the relation of the mouth with the rest of the body, that they have of a nail with the plank into which it is driven—and accordingly with such practical notions, they enter the mouth, and go to work upon the teeth, just as they would upon a block of granite, and with as little concern and knowledge for results, as a blacksmith has in the shoeing of a horse.

And without wishing, by any means, to do the slightest injustice, we would seriously ask if the above comparison is not in strict accordance with truth? And if so, the only legitimate and unavoidable inference for all such would-be dentists, seems to be, a wilful and total ignorance of the true character of the mouth and its several organs—being regarded by them simply as so many mechanical bodies, destitute, in every practical sense, of vitality, unorganized, and having little or no connection with the rest of the body, for any of the diseases to which they are subject.

Hence, how often does it occur, that patients have their teeth sacrificed in the hands of such dentists, when there was no disease present requiring such an operation—when, in fact, nothing was the matter with the teeth, and that the whole of the disturbance and pain was purely secondary or sympathetic, and dependent upon disease located either in the stomach or some other remote organ, and reflected to the teeth—in other words, the general relations of the body were primary in their dirturbance, and by their inseparable connection with those of the mouth, set up here a train of morbid symptoms in the teeth, flowing as the legitimate result of the *pathological relations* established between the whole system and the mouth, as one of its essential parts; hence, as there was no attempt to remedy the disease of the distant organ, which was the sole cause of the tooth-ache, the consequence was an entire failure in every case, and the pain continuing as violent as ever, notwithstanding the extraction of the teeth. And such cases, we are assured, from a conversation with Prof. Harris, are of frequent occurrence.

Now, had these dentists known that the teeth and all the parts belonging to the mouth were organized bodies—that they were endowed by the highest sensibilities, and vitalized by the same blood as every other organ—that they were equally dependent for their birth, growth and development upon the same common vascular and nervous system as the rest of the body—and that as an inseparable link in the same common chain, or as an essential part or series of parts in the great family or common brotherhood of organs—connected as the whole and each are, by relations, at once the most intimate, delicate and influential—and united as the whole are in one common cause—sharing in one common weal and woe—each dispensing and reciprocating the blessings of its own health to all the rest—and each alike sympathizing with and lightening as far as possible the sufferings of its afflicted brother organ, when laboring under disease—we say, with a knowledge of such relationship as this existing between the mouth and all the rest of the system, could these dentists justify their own consciences, or find any apology, unless it be the selfish one of gain, for the score of mutilations they have and are still constantly performing upon the mouths of their patients, for diseases which do not at all exist there, but are located elsewhere?

With these general remarks upon the too great indifference, neglect and ignorance, as manifested both by dentists and physicians, in reference to the reality—we mean the substantial and practical reality, of the *pathological relations* which the mouth has with every other part of the system, and the whole system has in turn with that of the mouth—we shall now proceed to notice more particularly the different *varieties* of the *pathological relations* of the *mouth*, with a view to demonstrating still more conclusively, and fortifying, if possible, still more strongly, the propositions already advanced.

### *Variety of Pathological Relations of the Mouth.*

A division of this class of relations has already been made, into *special* and *general.*

The *special variety* comprises all those diseases and morbid irregularities of the various organs belonging to the mouth, constituting the *dental apparatus*.

The organs included under this head are very numerous and important; so numerous as to present every variety of form, size, situation and structure—for here is seen bone, muscle, ligament, blood-vessel, nerve, gland, mucus, cellular structures, &c., in a word, all the anatomical elements found in any other part of the body; and, secondly, so important as to comprise in their functions both of the great divisions of the body, as made by Bichat, viz. the *animal* and *organic*.

The organs of the mouth, thus having the most extensive range, both in structure and function, and containing, as it were, a sample of every other structure and function, should, consequently, be liable to equally as great a diversity in the diseases to which they are subject, as all the rest of the body, both medical and surgical. And such is found to be the fact—the differences observed resulting rather from modifications of structure and function, which arise from the difference of arrangement in the same anatomical elements, and to differences of relation as to the various physical bodies with which all are in a greater or less degree connected. Hence we find inflammation, fever, suppuration, ulceration, fracture, dislocation, &c., are as common to the mouth and its various organs as the rest of the system, modified according to the simple differences just noticed.

Of the diseases of the organs of the mouth, arising from the special relations among themselves, it is only necessary here to simply give a brief summary of the principal, as these relations are generally admitted, and their diseases as generally recognized and acknowledged. We may mention, first, *caries* of a tooth, as frequently involving not only its own destruction, but likewise extending its diseased influence to that of its fibrous envelop and the alveolar socket—thence to the gums, and still onward to its neighbor teeth and the maxillary bones—producing inflammation, with all its consequences, &c., in all these various parts, if not arrested.

*Exostosis*, another form of diseased tooth, and consisting in an osseous excrescence found upon its root; it is well known, is not an unfrequent cause of disease in the antrum of Highmore or the maxillary sinus, as well as the cause also of most violent tooth-ache.

Even the *loss* of a tooth, it is asserted upon the best dental authority, is followed almost invariably by disease in that of its antagonist, thus seeming to show that constant mechanical pressure forms one of the laws necessary to dental health.

But this loss of one, and especially of several teeth, together with inflammation of the tonsils, and the congenital diseases of cleft palate, hare-lip, &c., not only extend their morbid influence to, but it is well known very materially derange all the functions of speech, prehension, suction, mastication and deglutition—functions most especially belonging to the dental apparatus. We will now trace diseases of the mouth a little further, and endeavor to show that the pathological relationship and morbid influence of the dental organs is by no means confined to themselves.

Dr. Koecker relates a very interesting case, and a very strong one to the point we wish to illustrate—and of which we can only give a very hasty outline:

It is the case of *tic doulourеux*, or neuralgia of the face, in a gentleman who had suffered "upwards of ten years" with this disease. The painful symptoms are described as unparalleled tortures, and "his whole constitution, but particularly the glandular system, was so much affected as to produce swellings and indurations in the most distant parts, accompanied with great pain and inconvenience; but its effects on his head were frequently agonizing; indeed, he assured me, so great were his sufferings, he had been so far driven to despair, as to implore heaven to relieve him, by putting an end to his miserable existence. He repeatedly applied for the best medical and surgical advice that the country could afford, but the real cause of his suffering was not detected; and such was the character of this disorder, that it baffled every exertion, and all the remedies which were applied for many years." A sea voyage and a trip to his native country were also tried in vain. At this period, the distinguished surgeon, Mr. Lawrence, was consulted, who, suspecting "the teeth to be the chief cause of his malady," recommended the patient to Dr. Koecker for the proper treatment.

The Dr. tells us he found "his gums and all his alveolar processes more or less diseased," and the teeth in such condition as to require the immediate extraction of thirteen. A gentle stimulant lotion to the mouth, and the removal of the tartar from the rest of the teeth, constituted the balance of the dental treatment, and we are told by the author of this case, that in less than one month the patient was "restored to perfect constitutional health."

In the July number, 1849, of the American Journal of Medical Sciences, several cases are reported by Dr.

Hays, of the effects of diseased teeth upon the eye. Our limits will only permit us to transcribe but a portion of one of the most interesting of these cases.

A gentleman, cashier in a bank of Wilmington, N. C., underwent, it seems, great fatigue, and taxed his eyes very severely, "in arranging the documents which were rescued from the flames," during an extensive fire in that place. "He soon experienced great intolerance of light, and some inflammation of the conjunctiva. For this he was treated by the physicians in his vicinity, but with only temporary relief, except for the inflammation. He subsequently visited Virginia and Raleigh, N. C., for medical advice; but from none of the remedies or plans of treatment employed in his case, did he experience the slightest permanent benefit; on the contrary, the photophobia increased to such a degree as to render exposure to the least light perfect torture." In this condition he arrived in Philadelphia, to consult Dr. Hays, who states that he found the patient "laboring under the most aggravated degree of photophobia. In a room so perfectly dark that the Dr. was unable to see any object whatever, to the patient the light reflected from his own hand was intolerable, and that from his shirt bosom caused so much suffering, that he was obliged to keep the latter constantly covered. So exalted was the sensibility of the retina, that in the darkened room, where Dr. H. could not see his hand held up before him, the patient was able to distinguish the objects around him, even the figures in the carpet." On examination, "scarcely a trace of inflammation of the eyes, or of any other apparent disease," was seen. The stomach is stated to have been a little deranged, and was corrected, without, however, any relief to the insufferable intolerance of light. The Dr. was now "induced to suspect the teeth," and it was found, on a careful examination, that several were "defective but not painful."

By his advice, a couple were extracted, but without giving any relief. On a second examination of the mouth, "upon striking one of the lateral upper incisores nearest to the eye most affected, with a key, the patient winced as from pain, and stated that he had often experienced a disagreeable sensation to proceed from that tooth. The tooth was extracted. With the loss of the tooth, a most disagreeable gnawing or pinching sensation at the back of the eye, which had previously tormented the patient, ceased. At the root of the tooth was found a large abscess, while the periosteum of the alveolus was thickened. From this time the morbid sensibilities of the eyes rapidly diminished, and the patient was soon after sufficiently recovered to return home, and resume his duties as cashier of the bank."

This case is doubly interesting and doubly instructive: First, to the dentist, in showing most conclusively that the organs which solely engage his attention, by no means limit their morbid influence to themselves, but extend their pathological relationship to other organs—and frequently, as in this case, producing in them a series of morbid secondary symptoms of the most violent character—and far more so than the primary set belonging to the teeth, the original seat and source of all the mischief. And further, practically instructive, as it teaches an earlier extraction than the very small amount of pain and visible disease of the teeth themselves would seem to warrant—and thus should lead us early most strongly to suspect derangement of these organs, and especially so, when medical treatment has failed to afford relief.

And, secondly, this case is particularly instructive to the physician, as it shows the too great neglect on his part, in overlooking the mouth, as the unsuspected but frequent cause of disease elsewhere situated—of disease entirely unmanageable without the knowledge and detection of this cause—but with this knowledge, and the application of proper dental treatment, found readily to yield. We say this case furnishes a solemn lesson to every physician—as it most plainly involves his own reputation, as to his requisite skill or great negligence in determining cause and effect—in other words, in finding out the real disease of his patient.

Jourdain and others have recorded inflammations of the eye, and the ear, as originating from morbid sympathies with the teeth; dyspepsia and gastric irritation also frequently arise from this source; and Dr. Rush speaks of having two cases, the one of *epilepsy* and the other of *rheumatism*, both of which were dependent upon diseased teeth, and that both were cured by their extraction.

Every one is familiar how the *irritation* from teething may be so violent upon the brain, as not unfrequently to cause delirium, terminating in convulsions, and it may be in death. Many other examples might be cited, but these are sufficient for our purpose, which is simply to show, under this head, that the pathological relations of the mouth are by no means limited to the dental organs themselves—but extend their morbid sympathies to other and distant parts—a *practical fact* which we humbly think cannot be too strongly impressed upon the attentive reflection of every dentist, physician and student—who is engaged, or expects to be engaged, in practice.

Now, the anatomical association of the whole of this class of morbid relations of the mouth—both of its own organs and those of distant ones, is mainly through the great fifth pair of nerves, which, by its own ramifications and anastomoses with other nerves, supply and connect all these various parts. It is true, the mucous membrane, blood-vessels and other structures, have no small share, also, in forming this common association.

We now pass to the second class of pathological relations of the mouth, which are perhaps still more important to the dentist than those just enumerated—but which, it may be safely said, the great mass not only do not recognize in their practice, but further, of whose real existence and value they seem to be most profoundly ignorant—and by such ignorance, we shall endeavor to show, they thus inflict all the evils of empiricism upon their deluded patients, as well as subject their own unblushing pretensions to the just judgment of constant failure—unless it be through sheer accident otherwise.

The relations we allude to are, viz. those which the *blood*, and *distant organs*, primarily exert upon the mouth.

And the disease we first notice, are those befalling the teeth during their formation—consisting in different degrees of density—presenting, thus, various shades of hardness and softness, and thereby forming so many morbid deviations from the natural healthy structure of these organs. Now, as the blood is the source from whence the teeth, as well as every other part of the body, derives the material both of its formation and development—it is manifest that if this blood be vitiated in its dental elements, that these, on being deposited in such a state, the teeth constituted of such diseased material, can by no possibility be constructed so as to present a sound and healthy structure.

Hence, to correct this evil, the dentist is obliged to correct the morbid condition of the blood, its source and origin; and this state of the blood may depend upon a diseased condition of the lungs—or upon impure chyle, which goes to form the blood; and this latter, again, upon a deranged state of the digestive organs, the agents of its formation—together with improper aliment, which may at the same time be taken into the system. Thus, then, it is seen what an extensive pathological relationship is involved in the production of the different morbid densities in the structure of the teeth above noticed—and that, consequently, to have a proper and scientific acquaintance with the true character of such diseased states of the teeth, as well as to indicate the proper plan of treatment for their correction—we say, to do this, it is absolutely necessary for every dentist to have at his command all the knowledge and all the resources of anatomy, physiology, pathology and practice, before he can intelligently trace cause and effect, and before he can intelligently apply the proper remedial measures—in a word, the studies of the dental student should not be a whit behind that of the medical—as the relations of the dental organs are coextensive with all the rest of the body, and consequently involve a knowledge of the whole, for the most scientific, successful and proper treatment of any particular part.

Again, *scurvy* is a disease of the blood, which, it is well known, makes its ravages principally upon the gums; now, no local application which the dentist can make to these parts, would be of any avail, unless the disease was met by proper constitutional treatment.

But it may here be said, that this disease does not come at all in the province of the dentist, and that he has nothing to do with it. We reply, that most certainly the gums belong to the dental organs, and as such, claim, and do receive the attention of all dentists, in their restoration to health when diseased; and though it is true, that this particular disease, scurvy, belongs properly to the physician for its treatment, yet how is the dentist to know this, should a man come into his office with such a disease of his gums, if he did not know that this was a constitutional disease—and if not knowing it, how then could he avoid the blunder, of immediately commencing a local treatment, by, probably, pulling out one or more of the teeth, as supposing them to be the cause of the disease of the gums, and thus mutilating the man's mouth, altogether unnecessarily—and showing, still further, in the highest degree, his ignorance, and the want of that skill and proper qualifications, which should characterise every honest, conscientious dentist, who attempts to practice the profession ?

The necessity of a general knowledge of the body to the dentist, with all its various relations, so as to avoid unnecessary operations upon the teeth, and its adjacent parts, which might otherwise be perpetually occurring, it may suffice, perhaps, in the way of further illustration, to simply enumerate a few more examples, without attempting any detail, as this article is already extended further than we at first anticipated.

We have, then,

1st. Tooth-ache, arising from an affection of the uterus.

2nd. Tooth-ache from gastric irritation.

3rd. Tooth-ache, imitating intermittent fever.

4th. Locked-jaw or trismus from a wound upon the extremities.

5th. Paralysis of the mouth, either partial or total, from disease of the brain or the seventh or fifth pair of nerves.

6th. Secretions upon the tongue and gums, of almost every color, viscidity and fetor—from fever of some form or other.

7th. Apthæ, from disordered stomach.

8th. Ulcers of the palate and other parts of the mouth, from syphilis—and so with numerous other diseases of distant organs, and those of a constitutional character—all alike furnish in greater or less degree so many examples of this general pathological relationship of the whole system, with the dental apparatus, as well as it is shown that it has with every other part.

In conclusion, if the propositions advanced, and the illustrations given in their support, in any way accord with the truth, it seems to necessarily follow, that every dentist, and every dental student should feel it his duty to thoroughly study the whole and every part of the body, and that too, both in health and disease, as the only trustworthy and safe foundation, upon which to rear an after superstructure in practice, that will combine at once all the harmony and beauty of scientific and moral proportions, and thus embody in its manly form, every element characterising the dentist of science and humanity, and thus fitting him, also, most efficiently, to go out on his high mission—of taking part with that noble profession, a member of which he certainly is, and whose common and divine art, alike consists in healing the sick.

